# Synchronous Multifocal Osteosarcoma: Case Report and Literature Review

**DOI:** 10.1155/SRCM/2006/53901

**Published:** 2006-09-04

**Authors:** Verity A. Currall, John H. Dixon

**Affiliations:** ^1^Department of Orthopaedics, Bristol Royal Infirmary, United Bristol Healthcare NHS Trust, Bristol BS1 3NU, UK; ^2^Avon Orthopaedics Centre, Southmead Hospital, North Bristol NHS Trust, Bristol BS16 1JE, UK; ^3^Department of Orthopaedics, Weston General Hospital, Weston-Super-Mare, North Somerset, BS23 4TQ, UK

## Abstract

*Background.* Multifocal osteosarcoma is usually described as the occurrence of the tumour at two or more sites in a patient without pulmonary metastases and may be synchronous or metachronous. *Case report.* A previously well 21-year old male, who presented with a swollen, painful right knee with no history of trauma, was found to have a high-grade osteosarcoma of the distal tibia and proximal femur. He underwent resection and prosthetic replacement of the distal femur and proximal tibia and remains well 19 months after diagnosis. *Discussion.* Multifocal osteosarcoma is a rare condition with a poor prognosis. There is debate about whether it represents multiple primary tumours or metastatic disease.

## BACKGROUND

Multifocal or multicentric osteosarcoma was first described by Silverman [1]. It is usually defined as the occurrence of the tumour at two or more sites in a patient without pulmonary metastases [2–4] and may be synchronous (more than one lesion at presentation) or metachronous (new tumours developing after the initial treatment). We present here a case of synchronous multifocal osteosarcoma with an unusual management dilemma and review of the literature.

## CASE REPORT

**Figure 1:**
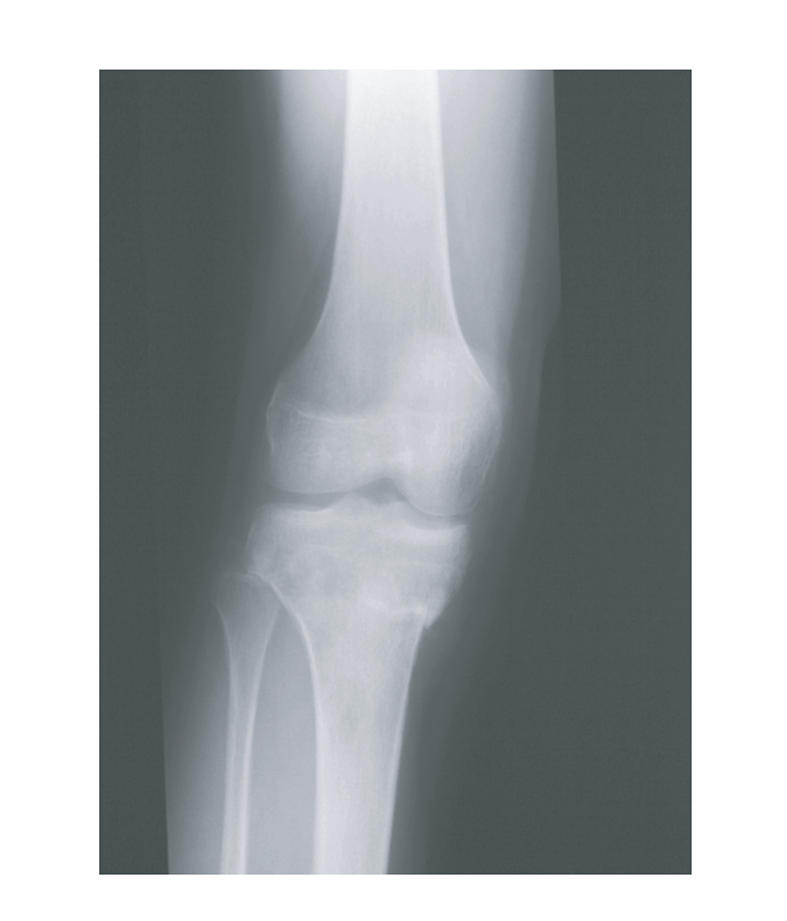
Pathological fracture of right proximal tibia.

The patient was a fit and well 21-year old man who initially presented to his GP with a painful right knee with no history of trauma. This was diagnosed as a soft tissue injury, but, six months later, he noticed a swelling in his right proximal tibia. A plain *x*-ray showed a pathological fracture of the proximal tibia (Figure 1) and he was referred to the regional sarcoma centre via his local fracture clinic.

**Figure 2:**
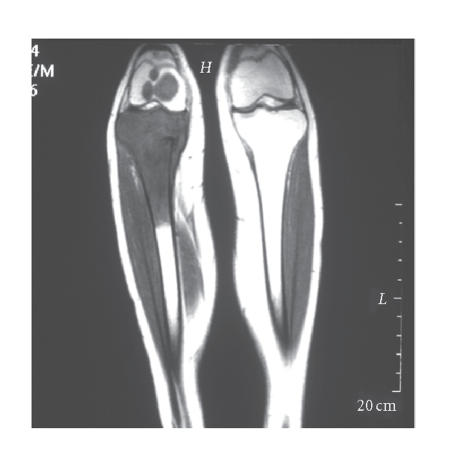
MRI right leg, showing osteosarcoma of proximal tibia with further lesions in the distal femur.

His initial investigations of blood tests, chest *x*-ray, and abdominal ultrasound showed no abnormalities, but MRI right knee clearly demonstrated an osteosarcoma in the proximal tibia with further lesions in the distal femur (Figure 2).

**Figure 3:**
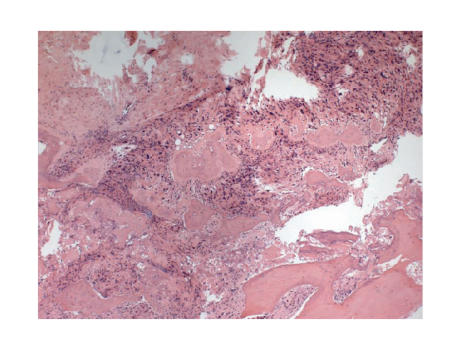
Biopsy of proximal femoral lesion showing high-grade intramedullary osteosarcoma.

A biopsy of the proximal tibial lesion confirmed the diagnosis of high-grade intramedullary osteosarcoma (Figure 3). Neoadjuvant chemotherapy of five cycles of doxorubicin and cisplatin was commenced.

**Figure 4:**
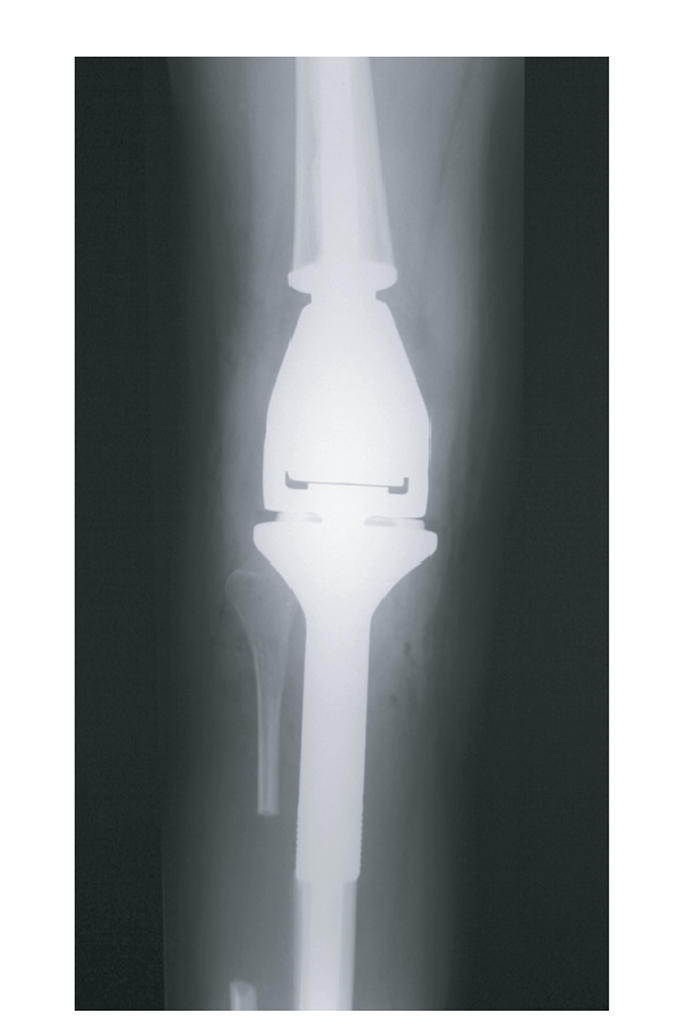
Postoperative *x*-rays showing prosthetic replacement.

The preoperative CT chest was clear and a further biopsy of both the distal tibial and proximal femoral lesions confirmed high-grade intramedullary osteosarcoma with necrosis. Because of this good response to chemotherapy, the decision was made to conserve the leg. Therefore, six months after diagnosis, the patient underwent resection of the right proximal tibia and distal femur with prosthetic replacement (Figure 4). The final histology showed necrotic bone tumour in the femoral and tibial lesions.

The patient underwent three months of postoperative chemotherapy and he remains clinically well. Nine months later, he was walking unaided and with knee flexion in excess of 90 degrees. There are no radiological signs of recurrence, either on plain *x*-ray or on MRI. A further MRI scan is planned shortly.

## DISCUSSION

Synchronous multifocal osteosarcoma is a rare condition, with a reported incidence of 1% to 3% [5–7]. There is much debate in the literature on whether it represents multiple primary tumours or metastatic disease. Initially, the case for multiple primary tumours was favoured [8, 9], because there was no obvious route for spread if the lungs were free of tumour, which was thought to rule out haematogenous metastasis. More recently, the report of cases related to p53 mutations [10] and retinoblastoma [11] might suggest a possible mechanism for multiple primary tumours.

However, more recent reviews conclude that the case for multicentric osteosarcoma as a metastatic process is almost proven [2, 12]. The reasons for this include the presence of a large “dominant” lesion, usually that leading to presentation, as in our case, which could be the primary tumour. Enneking and Kagan [13] suggest that bone-to-bone metastases could occur via a similar mechanism to that of prostate cancer via Batson's venous plexus [14], or intraosseous embolisation through marrow sinusoids. Alternatively, Hatori et al have demonstrated lymphatic spread to the lungs, giving another possible route [15]. It has also been noted that early case reports may well have underestimated the incidence of pulmonary metastasis, as the diagnosis of these relied on *x*-ray only, rather than CT scan [4]. Finally, there has been a correlation demonstrated between the responses of the dominant and other lesions to chemotherapy, which again points to a primary tumour and metastases [16].

The most commonly quoted classification is that of Amstutz [3], in which types I and II are synchronous (child/adolescent high grade and adult low grade, resp) and type III metachronous (subdivided into IIIa and b—early and late). Mahoney suggested a similar four-category system (A to D) ten years later [17]. They agree that the prognosis for synchronous multifocal osteosarcoma is poor, with mean survival of six months for type I/A and a slightly better range of 5 to 72 months for type II/B. Unfortunately, despite advances in both surgery and chemotherapy, more recent reports do not suggest a more favourable prognosis [7, 16, 18], with a mean survival of 27 months found by Bacci et al, although one patient was disease-free at nine years [16].

So what does this mean for our case? His has been a fairly standard course so far, although it could be argued that the pathological fracture predisposed to his multifocal presentation via either the venous or sinusoid routes. He remains alive and disease-free 19 months after diagnosis and will hopefully be one of the few who stays that way.
